# Smoking-Specific Parenting and Smoking Onset in Adolescence: The Role of Genes from the Dopaminergic System (DRD2, DRD4, DAT1 Genotypes)

**DOI:** 10.1371/journal.pone.0061673

**Published:** 2013-04-18

**Authors:** Marieke Hiemstra, Rutger C. M. E. Engels, Edward D. Barker, Onno C. P. van Schayck, Roy Otten

**Affiliations:** 1 Behavioural Science Institute, Radboud University Nijmegen, Nijmegen, The Netherlands; 2 Institute of Psychiatry, King's College, London, United Kingdom; 3 Care and Public Health Research Institute (CAPHRI), Maastricht University, Maastricht, The Netherlands; Yale University, United States of America

## Abstract

Although only few studies have shown direct links between dopaminergic system genes and smoking onset, this does not rule out the effect of a gene-environment interaction on smoking onset. Therefore, the aim of this study was to examine the associations between smoking-specific parenting (i.e., frequency and quality of communication and house rules) and smoking onset while considering the potential moderating role of dopaminergic system genes (i.e., DRD2, DRD4, and DAT1 genotypes). Data from five annual waves of the ‘Family and Health’ project were used. At time 1, the sample comprised 365 non-smoking adolescents (200 younger adolescents, mean age = 13.31, SD = .48; 165 older adolescents, mean age = 15.19, SD = .57). Advanced longitudinal analyses were used (i.e., logistic regression analyses, (dual) latent growth curves, and cross-lagged path models). The results showed a direct effect of quality of communication on smoking onset. No direct effects were found for frequency of communication and house rules. Furthermore, no direct and moderating effects of the DRD2, DRD4, or DAT1 genotypes were found. In conclusion, the findings indicated that the effects of smoking-specific parenting on smoking are similar for adolescent carriers and non-carriers of the dopaminergic system genes.

## Introduction

Tobacco use kills around six million people worldwide annually, being the leading preventable cause of death worldwide [1]. Still, thousands of young people start smoking every day. In 2011, 7% of the 10-year-old Dutch children tried smoking. This rate increased to 12% for 12-year-old children, 42% for 14-year-old children, and 63% for 18-year-old children [2]. Preventing tobacco use is important because early smoking is a strong predictor of developing long-enduring smoking habits [3]. Therefore, the present study focused on smoking initiation.

Behavioral genetic studies with twin designs have shown a significant genetic component of different stages of smoking [4]. The heritability has been estimated at 11–78% for smoking initiation, 28–84% for smoking persistence, and 50–58% for smoking cessation [4]. To investigate the genetic basis of smoking, molecular genetic studies have focused on specific genotypes (i.e., candidate gene studies). The central focus has been on the dopaminergic genes because of their role in the rewarding properties of nicotine [5]. The consumption of nicotine activates the mesolimbic dopamine system and increases dopamine release in the brain, resulting in feelings of pleasure or reward. The mesolimbic pathway transmits dopamine from the ventral tegmental area (VTA) of the midbrain to the nucleus accumbens. In the nucleus accumbens, nicotine increases dopaminergic activity [6]. The feeling of reward associated with the increase in dopamine release is one of the underlying mechanisms of the development of nicotine addiction. Activation of postsynaptic receptor neurons (i.e., dopamine receptor) and dopamine reuptake by presynaptic neurons (i.e., dopamine transporter) are important functions of the dopaminergic system. Ample studies have concentrated on genetic variations (polymorphisms) in three candidate genes, the dopamine receptors D2 (DRD2) and D4 (DRD4) as well as the dopamine transporter (DAT1).

The DRD2 is located on chromosome 11 and contains a TaqIA1 C>T polymorphism (rs 1800497). The DRD2 A1 allele has been associated with reduced dopamine D2 receptor availability and dopamine binding capacities in the brain, which may cause DRD2 A1 allele carriers to compensate for this reduced state of reward following the use of nicotine [7]. Several studies have examined the relationship between DRD2 A1 allele and smoking initiation. Three meta-analyses confirmed a small association between the DRD2 allele and smoking by reviewing 12, 13, and 21 studies [8]–[10]. Both reviews of Munafò et al. [9], [10] found limited but significant evidence for an effect of DRD2 on smoking initiation. Li and colleagues [8] found a significant relationship between DRD2 and smoking, although they did not specifically look at studies on the smoking initiation phenotype.

The DRD4 7-repeat allele of a 48-base-pair variable-number-of-tandem-repeats (VNTR) polymorphism in exon III is located on chromosome 11. The DRD4 plays an important role in nicotine craving [11]. Research showed that long (≥7 repeats of the DRD4) rather than short (<7 repeats) allele carriers are associated with decreased response to dopamine [12]. The long allele of the dopamine receptor gene has a lower potency to couple adenylyl cyclase, which is related to higher sensitivity to dopamine-related reward of nicotine [12]. To our knowledge, no reviews or meta-analyses examined the DRD4 genotype and smoking initiation. Gene-association research shows mixed results. For example, some studies have shown that the DRD4 long allele is associated with smoking [13]–[15]. Specifically, research indicates that African Americans with at least one long allele (6–8 repeats) started smoking at an earlier age and smoked more frequently compared to carriers of the short allele (2–5 repeats) [15]. This finding was replicated in a sample of European adolescents [13]. In addition, higher rates of smoking initiation were observed among those with long allele carries compared to those with other genotypes [14]. Nevertheless, other studies have reported no associations between DRD4 and smoking status (i.e., non-smoker, current smoker, and ex-smoker) [16]–[18]. In addition to the direct effects on smoking, effects of DRD4 on more indirect or proximal factors of smoking, such as smoking related cues have been shown [19].

The DAT1 transporter has a polymorphic 40-bp VNTR sequence located in the 3′ untranslated region, varying between 3 and 11 copies of which only 9- and 10-repeat alleles are common. The DAT1 is located presynaptically on dopaminergic neuron. It regulates the re-uptake of dopamine into presynaptic terminals, terminating dopaminergic neurotransmission and maintaining dopamine homeostasis [20]. The DAT-9 allele has been associated with a lower risk of early smoking onset and current smoking [21]–[24]. However, these findings have not been replicated [25], [26]. A meta-analysis of four studies [21], [22], [25], [26] by Munafò and colleagues [9] did not show that DAT1 was related to smoking initiation. However, a recent study by Ling and colleagues [27] on a single-nucleotide polymorphism (SNP) in the 3′-UTR of SCL6A3-9 (rs27072G4A) found that individuals with an A-allele were more likely to initiate smoking before the age of 18 compared to individuals without the A-allele. This result was not replicated in young adults [23]. Thus, the role of the DAT1 in smoking initiation has not been established.

In summary, studies focusing on direct effects of genes on the first stages of smoking showed mixed results. Evidence suggesting that direct genetic effects become more substantial in later stages of addiction is more convincing [28]. The absence of direct associations between genetic polymorphisms and smoking onset does not rule out the possibility that dopamine receptor and transporter genes relate to smoking initiation indirectly [29]. Specifically, it might be possible that genetic effects are present in early stages of smoking, such that environmental effects are stronger for children with certain genetic predispositions, implying gene-environment (GxE) interactions [30].

Parenting has been considered as an important environmental factor predicting smoking initiation [31]. Recently, research has shifted its focus from general parenting practices [31] to more proximal parenting behavior, i.e., anti-smoking socialization, as it might be easier for prevention and intervention programs to target such behavior [31]. Smoking-specific socialization comprises several parenting practices, such as discussing smoking related topics and setting rules not to smoke at home [32]. Previous research showed that constructive and respectful communication about smoking (i.e., quality of communication) prevents smoking initiation among adolescents [31], [33]–[35]. Divergent findings have been found for frequency of communication. Some studies found no effects or an increased likelihood of smoking initiation [34], [36] while others reported a decreased likelihood of smoking initiation [37]. Regarding smoking-specific house rules, a review of Emory and colleagues [38] revealed that house rules could prevent adolescents from starting to smoke.

Although low levels of smoking-specific parenting increase the likelihood of smoking initiation, adolescents vary in their response to these parenting practices, which might indicate the presence of GxE interactions [39]. The effects of smoking-specific parenting on adolescents with and without a genetic susceptibility to smoking initiation may differ. For instance, in a Finnish twin study, Dick and colleagues [40] found that parental monitoring protected vulnerable adolescents from smoking. Significant genetic influence on adolescent smoking decreased whereas common environmental influences increased with higher levels of parental monitoring. However, to date, no candidate gene studies examined gene-parenting effects on smoking initiation. Studies on alcohol use revealed the effect of the interaction between dopaminergic system DRD2 and parental rules [41], [42] while another study on cannabis use revealed the effect of the interaction between DRD4 and parental monitoring [43].

The present study examined the moderating effect of the dopaminergic system separately for different genotypes (i.e., DRD2 and DRD4 receptor genes and dopamine transporter gene DAT1) on the relation of specific aspects of smoking-specific parenting, such as frequency and quality of communication, smoking-specific house rules and smoking initiation using a five-wave prospective design. In order to concentrate on smoking onset, we only selected adolescents with no history of lifetime smoking. As adolescent smoking is a developmental process comprising different stages [44], parenting might also differ over time [33]. Therefore, longitudinal analyses were used to consider the development of parenting and adolescent smoking (i.e., development over time and bi-directional relations). We expected that DRD2, DRD4, and DAT1 genotypes would moderate the association between smoking-specific parenting and smoking onset of adolescents. We expected that low levels of smoking-specific parenting would only affect adolescents with a genetic susceptibility to smoking initiation.

## Methods

### Procedure

Data were drawn from five annual waves of the longitudinal Dutch ‘Family and Health’ study [34]. We selected 5062 addresses of families comprising father, mother, and two adolescents aged 13–16 years from 22 municipality registers. These families were invited to participate in the study. From 885 families that responded to the invitation and gave their informed consent, 765 met the inclusion criteria (i.e., parents were married or were living together and all family members were biologically related). Because of limited financial resources, we selected 428 families with an equal division of education and an equal amount of sibling dyads (i.e., 108 boy-boy, 118 boy-girl, 106 girl-girl, and 96 girl-boy).

Between November 2002 and April 2003 (time 1 = T1), an interviewer visited the families in their homes and asked each member of the family to complete a questionnaire. To ensure anonymity, respondents were asked to sit separately and avoid talking to each other about the questions. Subsequently, four annual follow-up interviews were conducted. Overall, 416 (time 2 = T2), 404 (time 3 = T3), 356 (time 4 = T4), and 326 (time 5 = T5) families participated at different time points, which reflects a high response rate of 76% over four years. Families received €30 per wave if all four family members completed the questionnaires. At T4, DNA samples were collected by means of saliva (Oragene; DNA Genotek Inc., Ottowa, ON, Canada). Overall, 311 families agreed to provide genetic data. Parental consent was obtained for all adolescents who participated. The independent medical ethics committee METiGG in Utrecht, the Netherlands (research 6209), approved the research design for this study.

Attrition analysis showed that genotyped adolescents (*n* = 622) had a higher educational level compared to non-genotyped adolescents (*n* = 234) (*OR* = 1.34, *95% CI* = 1.12–1.61, *p* = .001). The results indicated no differences between genotyped and non-genotyped adolescents in age, gender, adolescent smoking at T1, frequency of communication at T1, quality of communication at T1, and house rules at T1.

### Sample characteristics

At baseline, we only selected adolescents who never smoked and provided genetic data, resulting in 165 older adolescents and 200 younger adolescents. At baseline, older siblings were 14 to 16 years of age (M = 15.19, SD = .57) and younger siblings were 13 to 15 years of age (M = 13.31, SD = .48). Most adolescents were Dutch (Caucasian) (>96%). Boys and girls were represented almost equally, with 44.0% of the younger and 52.7% of the older adolescents being boys.

### Measures

#### Adolescent smoking

Smoking behavior of both adolescents was assessed at each wave. Adolescents reported the stage of smoking that applied to them on a nine-point scale ranging from 1 (I have never smoked, not even one puff) to 9 (I smoke at least once a day) [45]. For logistic regression, these responses were recoded to non-smoker = 0 (never smoking) and smoker = 1 (any experience with lifetime smoking) [34].

#### Maternal and paternal smoking

At each wave, parents reported the stage of smoking that applied to them using the same scale as was used by the adolescents [45]. However, one of the nine responses was less appropriate for them (i.e., ‘I tried smoking once in a while’); therefore, parents responded on an eight-point scale (cf. [34]). To address the skewness of the distribution of the data for eight categories and to establish a more robust measure of parental smoking, this variable was transformed to a new variable measured on a scale from 1 to 5 (1 = ‘I have never smoked, not even one puff’; 2 = ‘I tried smoking, I don't smoke anymore’; 3 = ‘I stopped smoking, after smoking at least once a month’; 4 = ‘I smoke occasionally, but not every day’; 5 = ‘I smoke at least once a day’) (cf. [35]).

#### Quality of smoking-specific communication

At each wave, the quality of communication was assessed using six items (per parent) reflecting a constructive and respectful way of communicating about smoking-related issues (e.g., ‘My mother/father and I are able to talk easily about our opinions concerning smoking’). Adolescents were asked to indicate answers that best applied to them on a 5-point scale ranging from 1 (completely not true) to 5 (completely true) [32]. Cronbach's alphas across waves ranged from .74 to .86 for the youngest adolescents and .80 to .88 for the oldest adolescents' reports about their mother and from .80 to .88 for the youngest adolescents and .84 to .87 for the oldest adolescents' reports about their father. Fathers' and mothers' quality of communication correlated highly (*r* = .75–.87, *p*<.001); therefore, we averaged the scale scores for father and mother (cf. [33]).

#### Frequency of smoking-specific communication

Frequency of communication was assessed at each wave by averaging the scores of eight items assessing how often parents talked with their child about smoking related issues in the past 12 months (e.g., ‘During the last 12 months, how many times did your mother/father talk to you about how to resist peer pressure to use tobacco?’) on a 5-point scale ranging from 1 (never) to 5 (very often) [32], [34], for an adapted Dutch version). Cronbach's alphas across waves ranged from .87 to .89 for the youngest adolescents and .86–.90 for the oldest adolescents' reports about their mother and from .89 to .91 for the youngest adolescents and .90–.91 for the oldest adolescents' reports about their father. For frequency of communication, the scores of parents correlated highly (*r* = .65–.75, *p*<.001); therefore, we averaged the scale scores (cf., [33]).

#### House rules

House rules were assessed at each wave by averaging the scores of five items assessing the existence of smoking-specific rules at home (e.g., ‘My parents and other adults are allowed to smoke indoors but children are not’, ‘At home, it is a rule that anyone who wants to smoke has to go outside’), which were measured on a 5-point scale ranging from 1 (completely not true) to 5 (completely true) [32]. Cronbach's alphas varied across waves, with values ranging from .74 to .82 for the youngest adolescents and .76 to .84 for the oldest adolescents.

### Genotyping

#### DRD2

The DRD2 TaqI A C>T polymorphism (rs1800497) was genotyped using Taqman analysis (assay ID: Taqman assay: C_7486676_10; reporter 1: VIC-A-allele, reverse assay; Applied Biosystems, Nieuwerkerk a/d IJssel, the Netherlands). Genotyping was conducted in a volume of 10 µl containing 10 ng of genomic DNA, 5 µl of Taqman Mastermix (2x; Applied Biosystems), .125 µl of the Taqman assay, and 3.875 µl of H_2_O. Genotyping was performed on a 7500 Fast Real-Time PCR System, and genotypes were scored using the algorithm and software supplied by the manufacturer (Applied Biosystems). To investigate the random genotyping error rate, the lab included five duplicate DNA samples per 96-wellplate, which were 100% consistent. In addition, four blanks, which were required to be negative, were included in each plate. Hardy–Weinberg equilibrium (HWE) proportions were estimated from parental genotype information using the Markov chain Monte Carlo approximation of the exact test implemented in the GENEPOP package version 3.3.54. No deviations from HWE were detected (*p* = .12). To maximize the power of the analyses, DRD2 genotype was dummy coded into 1 = non-risk (A2A2) and 2 = risk (A1A2 and A1A1) (cf. [10]).

#### DRD4

The 48-base-pair direct repeat polymorphism in *DRD4* was genotyped by amplifying 10 ng of genomic DNA in a 10-µl volume with the following components: .05 µM of fluorescently labeled forward primer VIC-5′-GCGACTACGTGGTCTACTCG-3′ (Applied Biosystems, Nieuwerkerk a/d IJssel, The Netherlands), reverse primer 5′-AGGACCCTCATGGCCTTG-3′, .4 mM of deoxynucleosidetriphosphates (dNTPs), and .5 U of La Taq (Takara, Lonza Verviers S.p.r.l., Verviers, Belgium). These were in a GC I buffer (Takara, Lonza Verviers S.p.r.l.) with 1 M betaine. The cycling conditions for amplification included 1 min at 94°C, 35 cycles of 30 s at 94°C, 30 s at 58°C, and1 min at 72°C, with an additional 5 min at 72°C. The length of the alleles was determined by direct analysis of an automated capillary sequencer (ABI3730, Applied Biosystems, Nieuwerkerk a/d IJssel, The Netherlands). HWE proportions were estimated. No deviations from these proportions were found (*p* = .87). Participants' *DRD4* genotype was dummy coded into 1 = non-risk (short allele, fewer than 7 repeats) and 2 = risk (7-repeat allele, at least one long allele) (cf. [13]).

#### DAT1

The 40-base-pair VNTR in the *SLC6A3* (*DAT1*) gene was genotyped, as described by Michelhaugh et al. [46]. Genomic DNA (62.5 ng) was amplified with .4 µM of forward primer (5′-TGTGGTGTAGGGACGGCCTGAGAG-3′), reverse primer (5′-CCTTGAGCCGTGACCTCCAGGAA-3′), and .25 mM dNTPs 0.5 U Taq DNA polymerase (Invitrogen) in a PCR buffer containing 60 mM Tris-HCl (pH 8.5), 15 mM (NH_4_)_2_SO_4_, 10% DMSO (v/v), and 3.5 mM MgCl_2_. The cycling conditions for the PCR assay started with 5 min at 92°C, followed by 35 cycles of 1 min 92°C, 1 min at 58°C, and 1 min 72°C, and additional 5 min 72°C. PCR products were analyzed on a 2% agarose gel, producing bands at 443 bp (9 repeats), 483 bp (10 repeats), or 523 bp (11 repeats). HWE proportions were estimated. No deviations from these proportions were found (*p* = .40). To maximize the power of the analyses, DAT1 genotype was dummy coded into 1 = non-risk (8/10, 10/10, and 10/11) and 2 = risk (9/9, 9/10, and 9/11) (cf. [21], [22]).

### Analyses

For the purpose of this study, we only included adolescents who had never smoked at baseline (*N* = 365; *n* = 200 younger adolescents and *n* = 165 older adolescents). The non-selected genotyped smokers (*n* = 254) were compared to the genotyped never smokers T1 (*n* = 365). Logistic regression analysis showed that genotyped lifetime smokers were older at baseline (OR = 1.42, 95% CI = 1.20–1.69, *p*<.001), were more likely to have fathers who smoked (OR = 1.20, 95% CI = 1.02–1.40, *p* = .03), talked more often about smoking (OR = 1.66, 95% CI = 1.27–2.17, *p*<.001), reported lower quality of communication (OR = .34, 95% CI = .25–.48, *p*<.001), and were more likely to carry the DRD4 non-risk allele (OR = .57, 95% CI = .39–.85, *p* = .005) compared to genotyped never smokers. After calculating descriptive statistics, we used three blocks of analyses, logistic regression analyses, latent growth curves (LGC), and cross-lagged modeling, to examine the associations between parenting and smoking onset and the moderating role of the dopamine DRD2, DRD4, and DAT1 genotypes. Logistic regression analyses were used to test the relation between parenting at T1 and smoking onset at T5. LGC were used to look at general trends of adolescent smoking (T2–T5) and parenting variables (T1–T5) over time. Cross-lagged modeling was used to assess specific relationships between parenting and smoking over time. These different statistical methods were used to ensure the integrity and robustness of our results.

Logistic regression analyses were conducted in SPSS 19.0 to examine the moderating effect of genes from the dopaminergic system on associations between parenting at baseline and smoking onset four years later. In the first step of the logistic regression analyses, we tested whether the covariates (i.e., age, gender, education, and smoking behavior of both parents) at T1 related to smoking status at T5. In the second step, a smoking-specific parenting variable at T1 and one dopamine gene were added to the model. In the third step, interaction terms between one parenting variable at T1 and a gene from the dopaminergic system (e.g., frequency of communication T1*DRD4) were entered.

Latent growth curves were used in the second block of the analyses to describe normative developmental patterns of behaviors. Significant variance in the growth curve parameters (i.e., intercept and slope) indicated that *individual* growth patterns deviated from the average growth patterns. Subsequently, we examined the effects of parenting at T1 on the intercept and slope of adolescent smoking and the moderating effect of dopamine genes on the link between parenting and adolescent smoking. Assuming that parenting also changes across time [33], we used growth models for two parallel processes (i.e., dual growth curves). We used the intercepts and slopes of the different parenting variables to predict the intercept and slope of adolescent smoking and the other way around. Multiple group analyses were used to examine the moderating effect of the dopamine genes (i.e., non-risk versus risk genotype). Differences in the intercept and slope parameters between the non-risk and risk genotypes were examined with a chi-square difference test by comparing a constrained model to the unconstrained model (i.e., intercept of adolescent smoking on the slope of parenting, and intercept of parenting on the slope of adolescent smoking).

**Figure 1:**
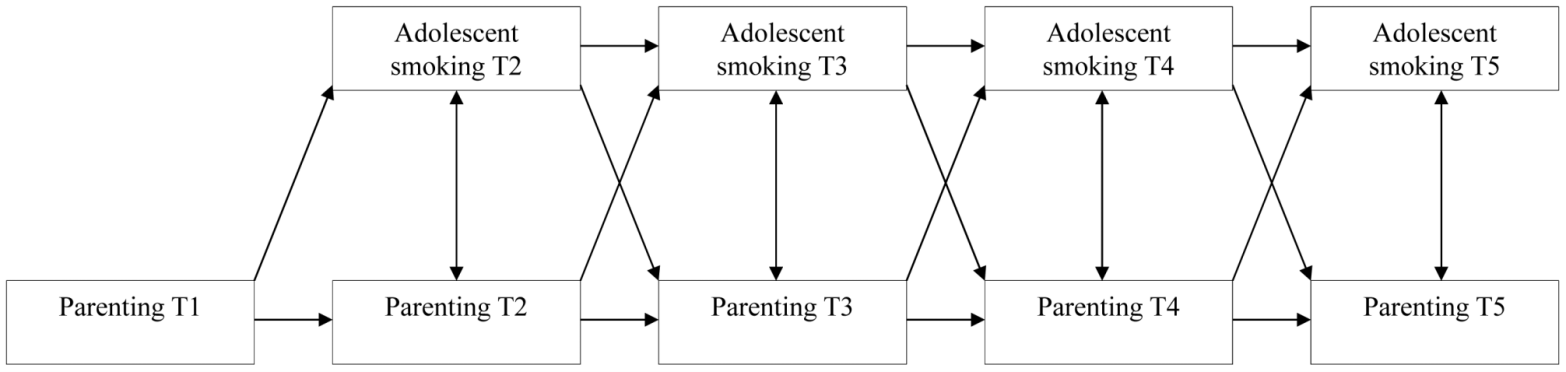
Cross-lagged path model for testing bi-directional relations between smoking-specific parenting (i.e. frequency and quality of communication and house rules) and adolescent smoking.

In the third and final block of analyses, we used cross-lagged modeling. Path analyses were used to test the longitudinal, bidirectional associations between each smoking-specific parenting strategy (T1–T5) and adolescent smoking (T2–T5) (see Figure 1) when controlling for covariates. A multigroup approach was used to examine the moderating role of the dopamine genes. To test the differences between the risk and non-risk genotype groups, different paths were constrained (i.e., stability paths, cross-lagged paths, stability and cross-lagged paths together). Differences in paths between the risk and non-risk genotype were examined again with a chi-square difference test.

Genetic effects of the DRD2, DRD4, and DAT1 polymorphisms were also examined using a cumulative genetic score (i.e., a combination of the different dopaminergic risk alleles) (cf. [47]). Each polymorphism was assigned value 1 if at least one risk allele was present. The values for each genetic polymorphism were added to create an index of cumulative genetic risk (0–3). The distribution was as follows: no (0) risk allele = 81 (22.8%); 1 risk allele = 166 (46.6%); 2 risk alleles = 89 (25.0%); and 3 risk alleles = 20 (5.6%). Category 2 and 3 were combined because of the group with 3 risk alleles was small. The 0 risk allele group was compared to the 1 risk allele group and with the 2/3 risk allele group. The results did not show significant differences between groups.

Our sample included both the oldest and youngest siblings of the 428 participating families. Our data were nested within families via the CLUSTER command in combination with TYPE = COMPLEX procedure in Mplus [48]. This method corrects for dependency that leads to unbiased standard errors of the estimated parameters. Because smoking was skewed, the parameters in the model were estimated using Maximum Likelihood estimator with robust standard errors (MLR). The MLR estimator yielded robust chi-square values, which were first rescaled to standard chi-square values before computing the chi-square difference test (i.e., the Satorra-Bentler scaled chi-square). The fit of the models was assessed using chi-square values *(df)*, Comparative Fit Index (CFI), Tucker-Lewis Index (TLI), and Root Mean Square Error of Approximation (RMSEA) [49]. Due to the number of tests, a Bonferonni correction was applied, and the results were adjusted to a *p*-value to≤.01.

## Results

### Descriptives and correlations

Proportions of adolescents ever smoking at T2 through T5 were 16.8%, 24.7%, 35.7%, and 39.1%, respectively. In total, 254 adolescents (69.8%) carried the non-risk DRD2 genotype (A2A2), 215 adolescents (59.2%) carried the non-risk DRD4 genotype (<7 repeats), and 209 adolescents (58.2%) carried the non-risk DAT1 genotype (other than 9 repeats).

Table 1 shows the means (standard deviations) of all variables and Pearson's correlations among them. Quality of communication was associated with lower smoking rates among adolescents at T2, T3, and T5 (−.12≤*r*≤−.19, *p*<.01). Frequency of communication and house rules were not associated with adolescent smoking. DRD2 and DRD4 genotypes were not associated with adolescent smoking, quality and frequency of communication, house rules, and parental smoking. DAT1 genotype was negatively associated with house rules (*r* = −.12, *p*<.01) and positively associated with frequency of communication (*r* = .17, *p*<.01) but not with adolescent smoking, indicating that the risk DAT1 genotype was associated with lower levels of house rules and higher levels of frequency of communication.

**Table 1:** Means (Standard deviations), Range and Pearson's Correlations among the study variables.

	*Mean (SD)*	*Range*	*1.*	*2.*	*3.*	*4.*	*5.*	*6.*	*7.*	*8.*	*9.*	*10.*	*11.*	*12.*
*1. Adolescent smoking T2*	1.38 (1.24)	1–9	*-*											
*2. Adolescent smoking T3*	1.70 (1.77)	1–9	.55***	-										
*3. Adolescent smoking T4*	2.13 (2.25)	1–9	.47***	.70***	-									
*4. Adolescent smoking T5*	2.37 (2.51)	1–9	.39***	.59***	.79***	-								
*5. Smoking mother T1*	2.59 (1.32)	1–5	.14**	.12*	.15**	.06	-							
*6. Smoking father T1*	2.58 (1.31)	1–5	.09	.15**	.13*	.09	.29***	-						
*7. House rules T1*	3.40 (1.02)	1–5	−.06	−.08	−.04	.01	−.38***	−.41***	-					
*8. Frequency of communication T1*	1.81 (.66)	1–5	.06	.07	.08	.02	.03	.04	.06	-				
*9. Quality of communication T1*	3.68 (.58)	1–5	−.19***	−.12*	−.08	−.13*	−.18**	−.21***	.14**	.19***	-			
*10. DRD4*	1.41 (.49)	1–2	−.08	.04	.02	.05	−.06	.03	.06	.02	.10	-		
*11. DRD2*	1.30 (.46)	1–2	−.08	−.05	−.05	−.08	.03	.05	−.03	.04	.004.	.02	-	
*12. DAT1*	1.42 (.49)	1–2	.05	.08	.01	.001	−.06	−.10	−.12*	.17**	.01	−.11*	.08	-

*Note*. Adolescent smoking: 1–9 scale, House rules: parental rules-setting from the adolescent's perspective; Frequency and quality of communication reported by the adolescent about father and mother (mean score of both); DRD2, DRD4, DAT1 genotype: 1 = non-risk, 2 = risk;

* *p*<.05,

** *p*<.01,

*** *p*<.001.

### Logistic regression analyses

Logistic regression analyses were used to examine the effects of smoking-specific parenting at T1 on lifetime smoking at T5 and the moderating roles of DRD2, DRD4, and DAT1 genotypes (see Table 2). No significant effects were found for the covariates (step 1) and parenting or the specific dopamine genes (step 2). Moreover, the analyses did not reveal significant interaction effects (step 3).

**Table 2:** Logistic regression analyses parenting at T1 (i.e., frequency of communication, quality of communication and house rules) predicting smoking onset at T5 and the moderating role of DRD2, DRD4 and DAT1 genotypes.

	*Frequency of communication*			*Quality of communication*			*House rules*		
	*DRD2*	*DRD4*	*DAT1*	*DRD2*	*DRD4*	*DAT1*	*DRD2*	*DRD4*	*DAT1*
	*OR _(95% CI)_*	*OR _(95% CI)_*	*OR _(95% CI)_*	*OR _(95% CI)_*	*OR _(95% CI)_*	*OR _(95% CI)_*	*OR _(95% CI)_*	*OR _(95% CI)_*	*OR _(95% CI)_*
*Step 1*									
Age	.91 _(.73–1.13)_	.93 _(.75–1.15)_	.91 _(.73–1.13)_	.91 _(.73–1.13)_	.93 _(.75–1.15)_	.91 _(.73–1.13)_	.91 _(.73–1.13)_	.92 _(.74–1.15)_	.90 _(.73–1.13)_
Gender	.72 _(.45–1.15)_	.70 _(.44–1.12)_	.73 _(.46–1.17)_	.72 _(.45–1.15)_	.70 _(.44–1.12)_	.73 _(.46–1.17)_	.72 _(.46–1.15)_	.70 _(.44–1.12)_	.73 _(.46–1.17)_
Education	.91 _(.70–1.18)_	.90 _(.69–1.17)_	.91 _(.70–1.18)_	.91 _(.70–1.18)_	.90 _(.69–1.17)_	.91 _(.70–1.18)_	.93 _(.71–1.22)_	.92 _(.70–1.20)_	.93 _(.71–1.22)_
Maternal smoking T1	1.04 _(.86–1.25)_	1.02 _(.85–1.23)_	1.01 _(.84–1.22)_	1.04 _(.86–1.25)_	1.02 _(.85–1.23)_	1.01 _(.84–1.22)_	1.04 _(.86–1.25)_	1.02 _(.65–1.23)_	1.01_(.84–1.21)_
Paternal smoking T1	1.10 _(.92–1.32)_	1.09 _(.91–1.31)_	1.13 _(.94–1.36)_	1.10 _(.92–1.32)_	1.09 _(.91–1.31)_	1.13 _(.94–1.36)_	1.10 _(.92–1.31)_	1.09 _(.91–1.31)_	1.13 _(.94–1.36)_
*Step 2*									
Parenting T1^1^	1.11 _(.78–1.57)_	1.07 _(.75–1.52)_	1.11 _(.78–1.57)_	.62*_(41–.95)_	.60*_(.39–92)_	.64*_(.42–.99)_	1.14 _(.89–1.47)_	1.21 _(.87–1.45)_	1.19 _(.91–1.55)_
Genotype^2^	.73 _(.44–1.22)_	1.35 _(.84–2.17)_	.99 _(.61–1.60)_	.74 _(.44–1.23)_	1.47 _(.91–2.39)_	1.01 _(.63–1.63)_	.74 _(.44–1.23)_	1.34 _(.84–2.16)_	1.08_(.67–1.75)_
*Step 3*									
Genotype* parenting T1	.60 _(.28–1.30)_	.61 _(.30–1.27)_	.97 _(.47–2.00)_	.83 _(.35–1.96)_	1.50 _(.64–3.47)_	.70 _(.31–1.60)_	.79 _(.48–1.30)_	1.06 _(.66–1.71)_	.87 _(.54–1.40)_

*Note*. gender: 1 = boy, 2 = girl; OR = Odds Ratio; 95% CI = 95% Confidence Interval;

* *p*<.05,

** *p*<.01,

*** *p*<.001.

1 please note that columns 1–3 refer to the predictor frequency of communication, columns 4–6 refer to quality of communication and 7–9 refer to house rules.

2 columns 1, 4, 7 refer to *DRD2* genotype, 2, 5, 8 refer to *DRD4* genotype and 3, 6, 9 refer to *DAT1* genotype.

### Latent growth curves

First, we calculated separate latent growth curves for adolescent smoking and parenting variables. The relative fit indices were satisfactory for all variables (see Table 3). We found no support for a quadratic trend. For adolescent smoking, frequency of communication, and house rules, the analyses revealed significant mean levels and significant inter-individual variability in the intercept and slope. For quality of communication, only the intercept was significant. However, both inter-individual variances of the intercept and slope were significant, indicating that although the mean level of quality of communication may be stable, some individuals indeed show significant changes in quality of communication over time.

**Table 3:** Model fit indices and growth curve parameters for adolescent smoking, frequency and quality of communication, and house rules.

Variable	χ^2^(*df*)	*p*	CFI	TLI	RMSEA	MeanIntercept	MeanSlope	VarianceIntercept	VarianceSlope
Adolescent Smoking	16.45 (5)	.006	.95	.94	.08	1.38SE = .07(19.40)***	.35SE = .04(8.10)***	1.14SE = .32(3.56)***	.49SE = .08(5.95)***
Frequency of communication (m/f)	17.95 (10)	.06	.98	.98	.05	1.78SE = .04(47.83)***	−.09SE = .01(−10.66)***	.29SE = .04(8.31)***	.01SE = .002(6.23)***
Quality of communication (m/f)	44.13 (10)	.00	.94	.94	.10	3.66SE = .03(121.16)***	−.01SE = .01(−1.21)	.22SE = .03(8.67)***	.02SE = .003(6.19)***
House rules	23.43 (10)	.01	.99	.99	.06	3.43SE = .07(52.12)***	.09SE = .01(6.75)***	.85SE = .06(13.72)***	.03SE = .006(4.53)***

*Note*. *T*-values are presented in parentheses below their respective associated growth curve parameter; CFI = Comparative Fit Index;

TLI = Tucker-Lewis Index; RMSEA = Root Mean Squared Error of Approximation;

*** *p*<.001,

** *p*<.01,

* *p*<.05, two-tailed tests.

Second, we examined the predictive value of parenting at T1 in relation to the intercept and slope of adolescent smoking and the moderating role of the dopamine genes (Table 4). The covariates were added in the first step, resulting in non-significant effects. In the second step, the dopamine genes and parenting at T1 were added. Subsequently, quality of communication related negatively to the intercept of smoking, indicating that higher quality of communication was associated with lower mean level of adolescent smoking. In step 3, we tested the interaction effects of DRD2, DRD4, and DAT1 genotypes, but none were significant.

**Table 4:** The effects of parenting at T1 on the intercept (I) and slope (S) of adolescent smoking.

	*DRD2*		*DRD4*		*DAT1*	
	Iβ (SE)	Sβ (SE)	Iβ (SE)	Sβ (SE)	Iβ (SE)	Sβ (SE)
*Step 1*						
Age	.03 (.05)	−.02 (.06)	.03 (.05)	−.02 (.06)	.03 (.05)	−.02 (.06)
Gender	.11 (.05)*	−.10 (.06)	.11 (.05)*	−.10 (.06)	.11 (.05)*	−.10 (.06)
Education	−.05 (.07)	−.08 (.06)	−.05 (.07)	−.08 (.06)	−.05 (.07)	−.08 (.06)
Maternal smoking T1	.14 (.09)	.01 (.06)	.14 (.09)	.01 (.06)	.14 (.09)	.01 (.06)
Paternal smoking T1	.08 (.09)	.06 (.06)	.08 (.09)	.06 (.06)	.08 (.09)	.06 (.06)
*Step 2*						
Genotype	−.09 (.04)*	−.06 (.06)	−.07 (.06)	.09 (.06)	.10 (.06)	−.01 (.06)
Frequency of communication T1	.08 (.06)	0.01 (0.06)	0.07 (.06)	.009 (.06)	.06(.06)	.01(.06)
Quality of communication T1	−.18 (.06)**	.04 (.06)	−.18 (.07)**	.03 (.67)	−.19 (.06)**	.03 (.06)
House rules T1	.02 (.08)	.06 (.07)	.02 (.07)	−.06 (.07)	.05 (.07)	.06 (.07)
*Step 3*						
Genotype* Frequency of communication	−.28 (.19)	−.23 (.22)	−.25 (.20)	−.36 (.23)	−.15 (.27)	.17 (.26)
Genotype*Quality of communication	.79 (.37)*	−.30 (.35)	.03 (.54)	−.33 (.41)	−.85 (51)	−.14(.39)
Genotype* House rules	.−.003 (.25)	−.31 (.23)	.33 (.23)	.04(.29)	.16 (.29)	−.06 (.25)

*Note*. SE = standard error; Gender: 1 = boys, 2 = girls; Model fits for full model of frequency of communication and DRD2:(χ^2^ = 39.99 (21), *p*<.001, CFI/TLI = .96/.94, RMSEA = .05), frequency of communication and DRD4: (χ^2^ = 42.15 (21), *p*<.01, CFI/TLI = .96/.93, RMSEA = .05) frequency of communication and DAT1: (χ^2^ = 39.57 (21), *p*<.01, CFI/TLI = .97/.94, RMSEA = .05), quality of communication and DRD2: (χ^2^ = 42.47 (21), *p*<.01, CFI/TLI = .96/.93, RMSEA = .05); quality of communication and DRD4: (χ^2^ = 45.08 (21), *p*<.01, CFI/TLI = .96/.92, RMSEA = .06); quality of communication and DAT1: (χ^2^ = 45.13 (21), *p*<.01, CFI/TLI = .96/.92, RMSEA = .06); house rules and DRD4: (χ^2^ = 38.65 (21), *p* = .01, CFI/TLI = .97/.94, RMSEA = .05); rules and DRD2 *(*χ^2^ = 35.70 (21), *p* = .05, CFI/TLI = .97/.95, RMSEA = .04); rules and DAT1: (χ^2^ = 38.78 (21), *p* = .01, CFI/TLI = .97/.94, RMSEA = .05),

*** *p*<.001,

** *p*<.01,

* *p*<.05.

Third, dual growth curves were calculated and the intercepts and slopes for the different parenting variables were used to predict the intercept and slope of adolescent smoking. The association between the initial values of quality of communication and adolescent smoking was significant (*β* = −.29, *p*<.001) (Table 5). Higher initial levels of adolescent smoking were associated with lower initial levels of quality of communication. Moreover, change in quality of communication was significantly associated with smoking initiation (*β* = −.48, *p*<.001). An increase in quality of communication over time related to a decrease in smoking initiation over time. Significant associations were found for initial values of parenting and change in parenting (frequency of communication: *β* = −.75, *p*<.001; quality of communication: *β* = −.28, *p*<.001; house rules: *β* = **−**.30, *p*<.001). Higher initial levels of parenting were associated with a decrease in parenting over time. Moderating effects of the DRD2, DRD4, or DAT1 genotypes were not found.

**Table 5:** Standardized estimates for the dual growth curve analyses between each smoking-specific parenting strategy and adolescent smoking controlled for age, gender, education, maternal and paternal smoking.

	*Frequency of communication* 1	*Quality of communication* 2	*House rules* 3
*Cross-lagged paths*			
S Smoking → I Parenting	.05(.07)	−.04 (.07)	−.004 (.06)
S Parenting → I Smoking	−.05 (.07)	.03 (.08)	.06(.10)
*Cross-sectional associations*			
I Smoking ↔ I Parenting	.08(.05)	−.29(.08)***	−.02 (.06)
S Parenting ↔ S Smoking	.18(.09)*	−.48 (.08)***	.18 (.08)*
*Stability associations*			
I Parenting ↔ S Parenting	−.75(.05)***	−.28 (.08)***	−.30 (.07)***
I Smoking ↔ S Smoking	.13 (.11)	.09 (11)	.10 (.10)

*Note I = Intercept, S = Slope*.

1 (χ^2^ = 95.45 (56), *p*<.01, CFI/TLI = .96/.94, RMSEA = .04).

2 (χ^2^ = 126.98 (56), *p*<.001, CFI/TLI = .95/.92, RMSEA = .06).

3 (χ^2^ = 90.59 (56), *p*<.01, CFI/TLI = .98/.97, RMSEA = .04);

*** *p*<.001,

** *p*<.01,

* *p*<.05.

### Cross-lagged path model

Cross-lagged models were tested to assess the causal relationship between three smoking-specific parenting strategies and adolescent smoking separately. The findings from these analyses are presented in Table 6. Concerning the quality of communication model, cross-lagged associations demonstrated that quality of communication was related to a decrease in smoking (T1 → T2: *b* = −19, *p*<.01; T3 → T4: *b* = −11, *p*<.01; T4 → T5: *b* = −14, *p*<.01). For frequency of communication and house rules, no significant cross-lagged paths were found. High stability paths were found for all three smoking-specific parenting strategies and smoking over time.

**Table 6:** Standardized estimates for the cross-lagged analyses between each smoking-specific parenting strategy and adolescent smoking controlled for age, gender, education, maternal and paternal smoking.

	*Frequency of communication* 1	*Quality of communication* 2	*House rules* 3
*Cross-lagged paths*			
Parenting T1 → Smoking T2	.06	−.19**	−.06
Parenting T2 → Smoking T3	.09	−.04	−.05
Parenting T3 → Smoking T4	.11*	−.11**	.06
Parenting T4 → Smoking T5	−.04	−.14**	−.01
Smoking T2 → Parenting T3	.07	−.07	.01
Smoking T3 → Parenting T4	.07	−.12*	.07
Smoking T4 → Parenting T5	.14*	−.01	.01
*Cross-sectional associations*			
Parenting T2 ↔ Smoking T2	.03	−.15*	.10
Parenting T3 ↔ Smoking T3	.003	−.13**	.01
Parenting T4 ↔Smoking T4	.14*	−.16**	.03
Parenting T5 ↔ Smoking T5	−.06	−.23***	.08
*Stability paths*			
Parenting T1 → Parenting T2	.59***	.56***	.76***
Parenting T2 → Parenting T3	.54***	.61***	.78***
Parenting T3 → Parenting T4	.56***	.62***	.76***
Parenting T4 → Parenting T5	.49***	.69***	.80***
Smoking T2 → Smoking T3	.56***	.55***	.56***
Smoking T3 → Smoking T4	.69***	.68***	.70***
Smoking T4 → Smoking T5	.80***	.75***	.80***

*Note.*

1 *χ^2^ = (df = 18, N = 365) = *69.50, *CFI/TLI = .95/.76, RMSEA = .*09;

2 *χ^2^ = (df = 18, N = 365) = *89.87, *CFI/TLI = .94/.74, RMSEA = .*11;

3 *χ^2^ = (df = 18, N = 365) = *117.17, *CFI/TLI = .95/.73, RMSEA = .*123;

*** ; *p*<.001,

** *p*<.01,

* *p*<.05.

Multi-group analyses were conducted to examine whether the cross-lagged paths differed for risk and non-risk genotypes. No significant decreases in fit were found by constraining stability paths, cross-lagged paths, or both stability and cross-lagged paths. This implies that the structural paths did not differ significantly between adolescents carrying non-risk or risk genotype.

## Discussion

The present study used advanced statistical techniques to test interactions between the dopamine receptor genes DRD2 and DRD4, dopamine transporter gene DAT1, and aspects of smoking-specific parenting (i.e., frequency and quality of communication, and smoking-specific house rules) on smoking initiation using a five-wave prospective design. In line with other studies, we found that constructive and respectful communication about smoking by parents could prevent children from smoking. No effects were found for frequency of communication, house rules, and smoking onset, as discussed elsewhere [33].

Furthermore, we did not find direct effects of the DRD2, the DRD4, and the DAT1 on smoking onset. This is in line with previous studies, which revealed weak effects of the DRD2 genotype and inconsistent evidence for the DRD4 and DAT1 genotypes [9], [10], [15]. As previous studies on alcohol use found an interaction effect between parenting and dopaminergic system genes [41], [42], we expected to find a similar interaction effect. However, we did not find any interaction effects of dopaminergic system genes with smoking-specific parenting, indicating that the relationships between smoking-specific parenting and smoking onset were similar for carriers and non-carriers of different genotypes.

One explanation for inconsistent findings regarding adolescent alcohol use and smoking may be that when it comes to smoking onset, peers are more influential than parents. Usually, the first experience with smoking cigarettes takes place in a peer context [50], whereas the first experience with drinking alcohol occurs more often at home, with parents [51]. Also, alcohol use is more embedded in the society compared to smoking cigarettes. In line with this reasoning, moderating effects of genes were found regarding the relationship between peer smoking and adolescent smoking [52]. A second explanation is that we focused on smoking initiation. It could be that the rewarding factor of the dopamine genes affects smoking initiation and other stages of smoking, such as smoking persistence and smoking cessation, differently [14]. Therefore, future studies should concentrate on more advanced stages of smoking and the possible interaction effects of dopamine genes and parenting. Third, divergent findings could also be due to studies utilizing different designs. Van der Zwaluw and colleagues [41] used prospective data with two time-points while Pieters and colleagues [42] utilized a cross-sectional design. The present study used five measurement waves and tested associations with time-varying estimates of parenting and smoking over time. Specifically, latent growth curve modeling and cross-lagged modeling were conducted with data collected at multiple time points, which provides richer data on the development of smoking and parenting compared to more traditional methods.

In addition to assessing the effect of a gene-environment interaction, we should consider possible gene-environment correlations. Parental genes can affect the child's environment (i.e., a passive gene-environment correlation), the child's genetic predisposition could affect parental behavior (i.e., a reactive gene-environment correlation), or the child seeking an environment conductive to their genetic predisposition (i.e., an active gene-environment correlation) [30]. These correlations may influence the effect of gene-environment interactions on the dependent variable [53]. In our study, we found a significant correlation of frequency of communication and house rules at T1 with the DAT1. However, we interpreted these results with caution, as the correlations were not consistent over time [i.e., only for frequency of communication at T1 (*r* = .17, *p*<.01) and T2 (*r* = .11, *p*<.05) and house-rules at T1 (*r* = −.12, *p*<.05) and T5 (*r* = −.14, *p*<.01)]. Furthermore, the DAT1 risk genotype was associated with less house rules, whereas it was positively associated with frequency of communication (i.e., higher levels of frequency of communication). It is important to stress that this is one of the first longitudinal studies, which followed adolescents throughout their teenage years, as most studies assessed smoking initiation retrospectively. In addition, we analyzed the potential effects of genotypes and subsequent interactions with smoking-specific parenting on smoking onset in various statistical models, assuring the consistency of the non-significant findings in this dataset.

Despite the robustness of our findings, some limitations should also be acknowledged. First, adolescents reported their own smoking behavior as well as smoking behavior of their parents. Although previous research has shown that self-reported data about smoking [54] are generally reliable, multi-informant data would have been preferable. Second, adolescents with a history of smoking at the first assessment were excluded from the analyses. The mechanisms that underlie smoking onset might differ for those who start early in adolescence and those who start in mid or late adolescence. Consequently, the results could not be generalized to younger adolescents or adults. Future research should study early smoking initiation among preadolescent children (i.e., 9–11 years old). Third, generalizability to the larger population was limited since we only included intact Dutch families with two children. Fourth, in this study, we examined only dopaminergic genes as genetic and parenting as environmental factors. Other genes, such as serotonin (e.g., 5-HTTLPR), opioid genes (e.g., OPRM1) [4] or other genes detected in genome-wide association studies (GWAS), may have an effect on smoking initiation [55]. In addition to parenting, individual factors, such as personality traits [56], may interact with genes. More research on different gene-environment interactions and smoking onset is required. Further, due to small effect sizes for a single polymorphism [57], more complex interactions may need to be investigated in the future (i.e., gene-gene interactions). For example, interactions between DAT1 and DRD2/DRD4 could be expected [21]. Finally, since the sample size in this study was relatively small, our findings should be replicated. On the other hand, a longitudinal design with measures at multiple waves increased the power of our study [58].

### Conclusion

We did not find evidence for a moderation effect of DRD2, DRD4, and DAT1 genotypes on the relationship between parenting and smoking onset. This indicates that parenting (i.e., quality of communication, frequency of communication, and house rules) affects carriers and non-carriers equally. To the best of our knowledge, this is the first study to examine the effects of parenting by dopaminergic system interactions on adolescent smoking. Therefore, replication is important. Future studies should attempt to increase our understanding of the interplay between genetic and environmental risk factors on smoking onset and provide recommendations for future prevention programs.
